# Nephroprotective Effects of Semaglutide as Mono- and Combination Treatment with Lisinopril in a Mouse Model of Hypertension-Accelerated Diabetic Kidney Disease

**DOI:** 10.3390/biomedicines10071661

**Published:** 2022-07-11

**Authors:** Louise S. Dalbøge, Michael Christensen, Martin Rønn Madsen, Thomas Secher, Nicole Endlich, Vedran Drenic’, Alba Manresa-Arraut, Henrik H. Hansen, Ida Rune, Lisbeth N. Fink, Mette V. Østergaard

**Affiliations:** 1 Gubra, DK-2970 Hoersholm, Denmark; mch@gubra.dk (M.C.); mam@gubra.dk (M.R.M.); txse@novonordisk.com (T.S.); ama@gubra.dk (A.M.-A.); hbh@gubra.dk (H.H.H.); irs@gubra.dk (I.R.); lnf@gubra.dk (L.N.F.); 2 Center of High-End Imaging, NIPOKA GmbH, 17489 Greifswald, Germany; nendlich@nipoka.com (N.E.); vdrenic@nipoka.com (V.D.); 3 Vifor (International) Ltd., 9001 St. Gallen, Switzerland; mette.ostergaard@viforpharma.com

**Keywords:** diabetic kidney disease, hypertension, mouse model, glomerulosclerosis, podocyte, GLP-1 receptor agonist, ACE inhibitor

## Abstract

**Background**: Obesity, hyperglycemia and hypertension are critical risk factors for development of diabetic kidney disease (DKD). Emerging evidence suggests that glucagon-like peptide-1 receptor (GLP-1R) agonists improve cardiovascular and renal outcomes in type 2 diabetes patients. Here, we characterized the effect of the long-acting GLP-1R agonist semaglutide alone and in combination with an ACE inhibitor (lisinopril) in a model of hypertension-accelerated, advanced DKD facilitated by adeno-associated virus-mediated renin overexpression (ReninAAV) in uninephrectomized (UNx) female diabetic *db*/*db* mice. **Methods**: Female *db*/*db* mice received a single intravenous injection of ReninAAV 1 week prior to UNx. Six weeks post-nephrectomy, *db*/*db* UNx-ReninAAV mice were administered (q.d.) vehicle, semaglutide (30 nmol/kg, s.c.) or semaglutide (30 nmol/kg, s.c.) + lisinopril (30 mg/kg, p.o.) for 11 weeks. Endpoints included blood pressure, plasma/urine biochemistry, kidney histopathology and RNA sequencing. **Results**: Vehicle-dosed *db*/*db* UNx-ReninAAV mice developed hallmarks of DKD characterized by severe albuminuria and advanced glomerulosclerosis. Semaglutide robustly reduced hyperglycemia, hypertension and albuminuria concurrent with notable improvements in glomerulosclerosis severity, podocyte filtration slit density, urine/renal kidney injury molecule-1 (KIM-1) levels and gene expression markers of inflammation and fibrogenesis in *db*/*db* UNx-ReninAAV mice. Co-administration of lisinopril further ameliorated hypertension and glomerulosclerosis. **Conclusions**: Semaglutide improves disease hallmarks in the *db*/*db* UNx-ReninAAV mouse model of advanced DKD. Further benefits on renal outcomes were obtained by adjunctive antihypertensive standard of care. Collectively, our study supports the development of semaglutide for management of DKD.

## 1. Introduction

Diabetic kidney disease (DKD) is a long-term complication of diabetes and the leading cause of end-stage kidney disease worldwide. The clinical manifestation of DKD is characterized by progressive loss of kidney function. In addition to hyperglycemia, several metabolic risk factors can predispose to DKD, including hypertension, obesity, and dyslipidemia [1]. Accordingly, controlling blood glucose and lowering blood pressure is a cornerstone in the prevention and management of DKD. For the past decades, blockade of the renin-angiotensin system (RAAS) using angiotensin-converting enzyme (ACE) inhibitors and angiotensin II receptor antagonists has been the standard of antihypertensive care which improve both renal and cardiovascular outcomes in patients with type 2 diabetes (T2D) [2,3]. Beyond the glycemic effects of sodium-glucose co-transporter-2 (SGLT2) inhibitors, this class of glucose-lowering agents has recently shown beneficial renal and cardiovascular effects to reduce the onset and progression of CKD in patients with and without diabetes [4,5,6]. Another class of glucose-lowering drugs, glucagon-like peptide-1 receptor (GLP-1R) agonists, is emerging as a promising treatment option in DKD. GLP-1 peptide-based therapies represent a major advance in the treatment of obesity and T2D, promoting an unprecedented weight loss and substantial improvements in glycemic control [7]. Interestingly, recent cardiovascular outcome trials on GLP-1R agonist treatment in diabetic patients have indicated delay in the onset and progression of CKD [8,9,10]. The long-acting GLP-1 analogue, semaglutide, which is currently approved for the treatment of T2D and obesity [11,12], has been demonstrated to improve cardiorenal outcomes in patients with T2D [9,10]. Notably, the current FLOW semaglutide trial (clinicaltrials.gov, #NCT03819153) is the first to directly address the efficacy of a long-acting GLP-1R agonist on primary endpoints in progressive DKD. While initial data from the cardiovascular outcome trials are encouraging, further studies are needed to substantiate the renoprotective effects and mode of action of GLP-1R agonists.

A state-of-the-art translational mouse model of hypertension-accelerated DKD has recently been established, induced by adeno-associated virus (AAV)-mediated renin overexpression in the uninephrectomized (UNx) diabetic *db*/*db* (*db*/*db* UNx-ReninAAV) mouse [13,14,15]. *db*/*db* UNx-ReninAAV mice display key features of advanced diabetic kidney disease, including extreme albuminuria and severe glomerulosclerosis, which can be ameliorated by current clinical standard of care using ACE inhibitor (lisinopril) and SGLT2i (empagliflozin) combination therapy [13]. To gain further insight into the nephroprotective effects of GLP-1R agonists, we characterized therapeutic outcomes of semaglutide as monotherapy and with lisinopril co-administration in *db*/*db* UNx-ReninAAV mice.

## 2. Materials and Methods

### 2.1. Animals

The Danish Animal Experiments Inspectorate approved all experiments which were conducted using internationally accepted principles for the use of laboratory animals (license #2013-15-2934-00784). Female *db*/*db* (BKS.Cg-Dock7m+/+Leprdb/J) mice (5 weeks old) were obtained from Charles River (Calco, Italy) and housed in a controlled environment (12:12 light/dark cycle, lights on at 3 a.m., 23 ± 2 °C, humidity 50 ± 10%). Female *db*/*db* mice are less prone to develop hydronephrosis and pyelonephritis with increasing age compared to males [16,17,18]. Each animal was identified by an implantable subcutaneous microchip (PetID Microchip, E-vet, Haderslev, Denmark). Mice had ad libitum access to standard chow (Altromin 1324, Brogaarden, Hørsholm, Denmark) and tap water.

### 2.2. Uninephrectomy and Adeno-Associated Virus Delivery of Renin

**Figure 1:**
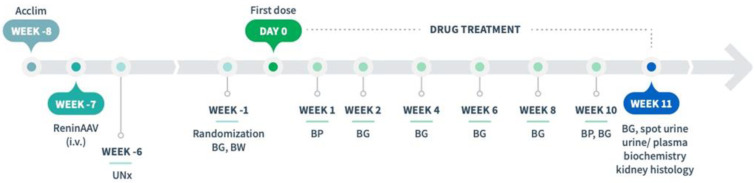
Study outline. *db*/*db* UNx-ReninAAV mice were treated (q.d.) with vehicle (s.c.), semaglutide (30 nmol/kg, s.c., or combination treatment with semaglutide (30 nmol/kg, s.c.) + lisinopril (30 mg/kg, p.o.) for 11 weeks. Abbreviations: AAV, adeno-associated virus; Acclim., acclimatization; BG, blood glucose; BP, blood pressure; BW, body weight; i.v., intravenous; UNx, uninephrectomy.

An adeno-associated virus (AAV) construct AAV8-TBG-m-Ren1d (F61R/P65S, Vector Biolabs, Malvern, PA), expressed under the control of the liver-specific thyroxin-binding globulin promotor [13,19], was used to induce hypertension [14,20]. ReninAAV (2 × 10^10^ genome copies, GC) was suspended in sterile PBS and administered by tail vein injection in conscious animals (6 weeks of age). One week after AAV injection, UNx was performed as described previously [14,18], see Figure 1. Animals were allowed to recover for 6 weeks before study start (defined as Day 0). Body weight was measured twice a week from one week before AAV administration until study start, thereafter once daily.

### 2.3. Drug Treatment

Treatment was initiated 7 weeks after AAV administration and 6 weeks post-UNx (Figure 1). Randomization and stratification to treatment was based on fed blood glucose and body weight measured one week before treatment start. *db*/*db* UNx-ReninAAV mice (*n* = 14–15 per group) received (q.d.) vehicle (0.5% methyl cellulose, s.c.), semaglutide (30 nmol/kg, s.c., Bachem AG, Bubendorf, Switzerland), or combined semaglutide (30 nmol/kg, s.c.) + lisinopril (30 mg/kg, p.o., MedChemExpress, Monmouth Junction, NJ, USA) for 11 weeks. A dose-escalation scheme was implemented to reduce expected initial effects of semaglutide treatment, as transient GLP-1R-induced discomfort in rodents, including taste aversion and pica behavior, is typically observed within the first 2–3 days of treatment [21].

### 2.4. Blood Pressure

Diastolic, systolic and mean arterial blood pressure were measured in treatment week 1 and 10 by tail cuff plethysmography (CODA, Kent Scientific, Torrington, CT, USA).

### 2.5. Blood and Urine Analyses

Blood and urine samples were collected from non-fasted animals during the light phase. Tail blood samples were collected every 2 weeks for blood glucose analysis. Terminal blood samples were analyzed for blood glucose and glycated hemoglobin A1c (HbA1c) as well as plasma urea and cystatin C [14,18]. Terminal spot urine was assayed for albumin, creatinine and kidney injury molecule-1 (KIM-1, R&D Systems, Minneapolis, MN, USA) as described previously [14,18].

### 2.6. Histomorphometry

Histology was performed on sections from formalin-fixed kidneys as described in detail elsewhere [18,22]. Stained sections were scanned with a 20× objective using a Scanscope AT slide scanner (Aperio, Leica Biosystems, Buffalo Grove, IL, USA) and quantitative image analysis was performed using Visiomorph software (Visiopharm, Hørsholm, Denmark). KIM-1 (AF1817, R&D systems, Minneapolis, MN, USA) was quantified in whole-kidney sections, expressed as proportional (%) surface area of immunopositive staining.

### 2.7. Glomerulosclerosis Scoring Using Deep-Learning Computational Analysis

Glomerulosclerosis scoring was performed by AI-assisted image analysis as described previously [14]. In brief, slides were scanned with a 20× objective and image analysis was performed with Python. Glomerulosclerosis score was computed by detecting all glomeruli using U-net network architecture [23], whereafter each glomerulus was assigned a glomerulosclerosis score using inceptionv3+ network architecture [24]. Glomeruli were classified according to a five-point scale using the following criteria: GS0 (normal), GS1 (mild, sclerotic area up to 25%), GS2 (moderate, sclerotic area 25–50%), GS3 (severe, sclerotic area 51–75%) and GS4 (global, sclerotic area 76–100%). Data were expressed as the number of glomeruli with individual glomerulosclerosis score (GS0-GS4 and GS3-GS4, respectively) relative to total glomeruli counts in the corresponding experimental group (fraction %). The glomerulosclerosis index was calculated using the following formula: (1 × n1) + (2 × n2) + (3 × n3) + (4 × n4)/n0 + n1 + n2 + n3 + n4, where nx was the number of glomeruli in each grade of glomerulosclerosis [25].

### 2.8. Filtration Slit Density Analysis

After deparaffinization and rehydration, kidney sections (2 µm) were boiled in Tris-EDTA buffer (10 mmol/L Tris, 1 mmol/l EDTA, pH 9) in a pressure cooker for 5 min, followed by the blocking step (1% FBS, 1% BSA, 0.1% fish gelatine, 1% normal goat serum) for 1 h. The following primary antibodies were incubated overnight at 4 °C: rabbit anti-podocin 1:150 (IBL International, Hamburg, Germany) and mouse anti-synaptopodin 1:75 (Progen, Heidelberg, Germany). After washing three times in PBS, secondary antibodies were incubated for 1 h at room temperature (anti-rabbit Alexa Fluor 488-conjugated IgG 1:1200 (ChromoTek, Planegg, Germany) and anti-mouse Cy3-conjugated IgG antibody 1:600 (Jackson ImmunoResearch, West Grove, PA, USA) for 1 h at room temperature. DAPI (1:100) was added to the slides for 5 min, followed by a washing step in PBS. Finally, the slides were incubated in H_2_O and mounted in Mowiol (Carl Roth, Karlsruhe, Germany) using high-precision coverslips (Paul Marienfeld, Lauda-Königshofen, Germany). The evaluation of the filtration slit density was performed using a recently established super-resolution microscopy-based methodology (structured illumination microscopy) termed as podocyte exact morphology measurement procedure (PEMP) [26]. For three-dimensional structured illumination microscopy (3D-SIM), z-stacks of 19 planes of both channels (488 and 561 nm) were acquired from the stained kidney section using an N-SIM super-resolution microscope (Nikon, Tokyo, Japan) equipped with a 100× silicone objective. The images were reconstructed into 3D-SIM images using NIS-Elements AR 5.30 (Nikon). The z-stacks were converted into a maximum intensity projection followed by the automatized identification of the filtration slit length as an index of foot process effacement. The peak-to-peak distance of two neighbouring filtration slits was measured on the half length of a single foot process from its origin from the major process to its tip. The filtration slit density (FSD) was expressed as the ratio of the total filtration slit diaphragm per podocyte foot process area. The FSD of 20 glomeruli was determined for every animal.

### 2.9. RNA Sequencing

Transcriptomics was performed using RNA sequencing on RNA extracts from kidney cortex-enriched samples (15 mg fresh tissue), as described in detail elsewhere [27]. RNA sequence libraries were prepared with NeoPrep (Illumina, San Diego, CA, USA) using Illumina TruSeq stranded mRNA Library kit for NeoPrep (Illumina, San Diego, CA, USA) and sequenced on the NextSeq 500 (Illumina, San Diego, CA, USA) with NSQ 500 hi-Output KT v2 (75 CYS, Illumina, San Diego, CA, USA). Reads were aligned to the GRCm38 v84 Ensembl Mus musculus genome using STAR v.2.5.2a with default parameters. A lower detection limit for gene expression was defined based on raw mapped read counts (RPKM of 0.1). The R-package DESeq2 was used for differential gene expression analysis. Gene set analysis was conducted with the R package PIANO version 1.18.1 using the Stouffer method, and *p*-values were corrected for multiple testing using the Benjamini–Hochberg method (5% false discovery rate, FDR < 0.05). To obtain further resolution of gene expression regulations, expression of treatment-regulated genes was evaluated across kidney cell types using single-nuclei sequencing data from *db*/*db* UNx-ReninAAV mice, with data processed as described previously [15]. For clustering analysis, the geometric mean of relative counts +1 was calculated for each cell type, and mean values were centred and scaled prior to hierarchical clustering.

### 2.10. Statistics

Except for RNA sequencing, data were analysed using GraphPad Prism v.9.0.2 software (GraphPad, La Jolla, CA, USA). All results are shown as mean ± standard error of mean (SEM). Dunnett’s test one-factor/two-factor linear model with interaction were used with a *p*-value < 0.05 considered statistically significant. For filtration slit density analysis, normality of the groups was tested using Shapiro–Wilk normality test. Groups were compared using one-way ANOVA corrected for multiple comparison by controlling FDR using the method of Benjamini and Hochberg. FDR-adjusted *p*-values of less than 0.05 were considered significant.

## 3. Results

### 3.1. Semaglutide Improves Obesity and Hyperglycemia in db/db UNx-ReninAAV Mice

**Figure 2:**
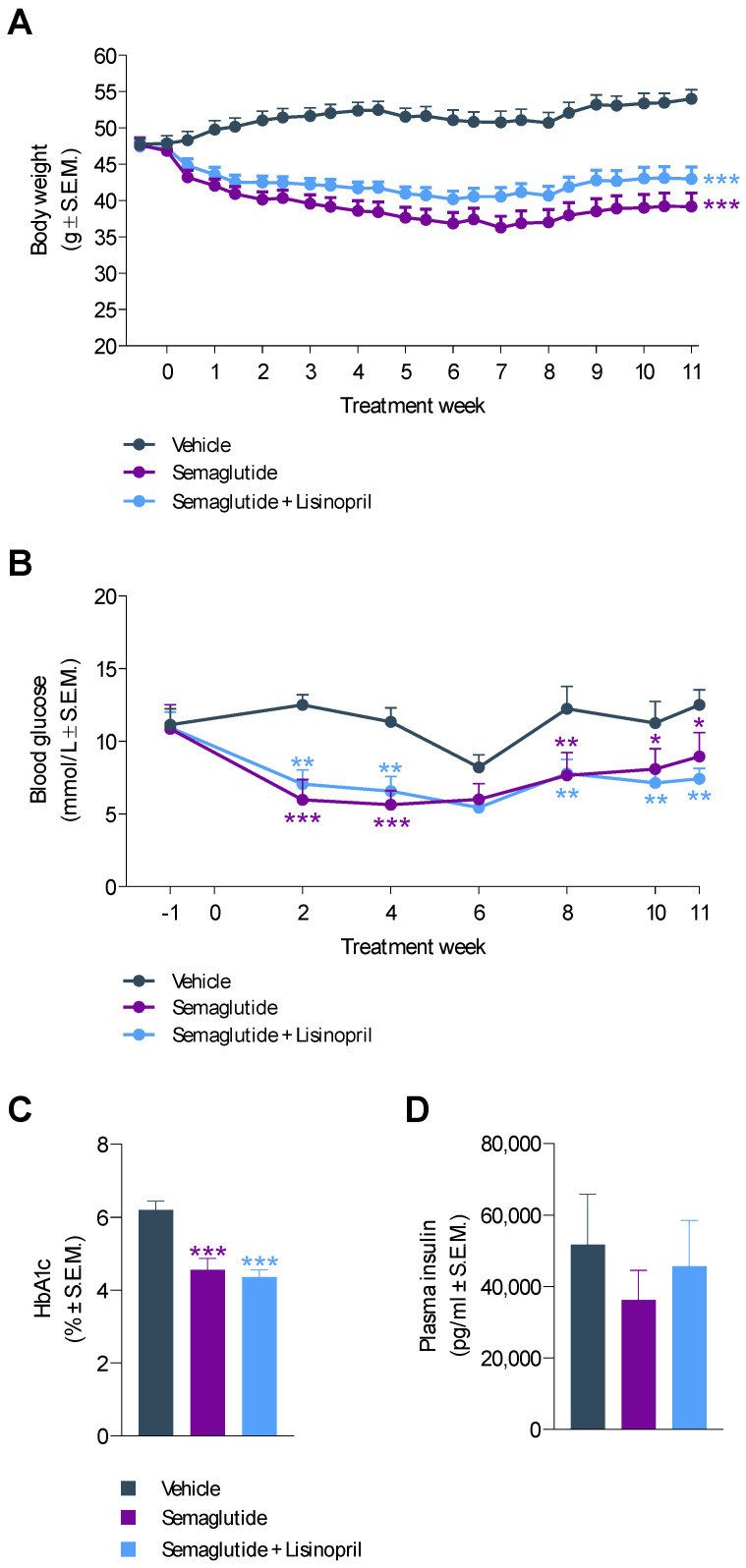
Semaglutide reduces body weight and improves hyperglycemia in *db*/*db* UNx-ReninAAV mice. (**A**) Body weight. (**B**) Fed blood glucose levels. (**C**) Terminal HbA1c levels. (**D**) Terminal plasma insulin concentrations. Semaglutide monotherapy (30 nmol/kg, s.c., q.d., *n* = 15) and combination treatment with lisinopril (30 mg/kg, p.o. q.d., *n* = 14) was performed for 11 weeks in *db*/*db* UNx-ReninAAV mice. Vehicle-dosed (s.c., q.d.) *db*/*db* UNx-ReninAAV mice (*n* = 15) served as controls. * *p* < 0.05, ** *p* < 0.01, *** *p* < 0.001 vs. vehicle-dosed *db*/*db* UNx-ReninAAV mice (Dunnett’s test one-factor/two-factor linear model with interaction).

Compared to baseline, a sustained weight loss was observed after 11 weeks of semaglutide (−16.2 ± 3.0%) and semaglutide + lisinopril (−8.2 ± 3.5%) treatment in *db*/*db* UNx-ReninAAV mice (Figure 2A). The slower decline in body weight with semaglutide + lisinopril treatment did not attain statistical significance when compared to semaglutide monotherapy (*p* = 0.126). In contrast, vehicle controls showed progressive weight gain over the entire course of the study compared to baseline (13.8 ± 2.5%, *p* < 0.001) (Figure 2A). Semaglutide and semaglutide + lisinopril improved hyperglycemia to a similar extent, being sustained after two weeks of treatment (Figure 2B). Improved glycemic control was further supported by significantly lowered HbA1c levels following long-term semaglutide and semaglutide + lisinopril treatment, respectively (Figure 2C). Treatments had no effect on terminal fed insulin levels (Figure 2D).

### 3.2. Semaglutide Improves Hypertension in db/db UNx-ReninAAV Mice

**Figure 3:**
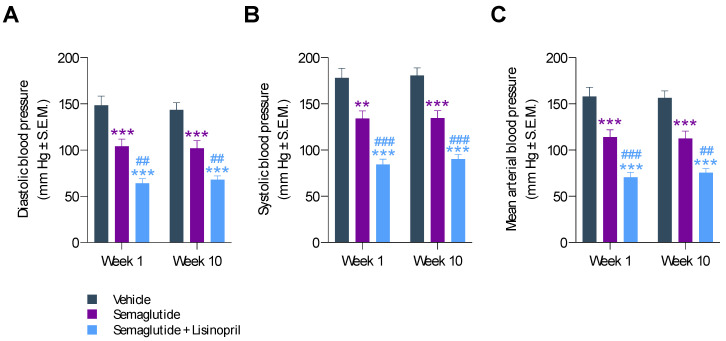
Semaglutide improves hypertension in *db*/*db* UNx-ReninAAV mice. (**A**) Diastolic arterial blood pressure. (**B**) Systolic arterial blood pressure. (**C**) Mean arterial blood pressure. Blood pressure was measured by tail cuff plethysmography in treatment week 1 and 10. Semaglutide monotherapy (30 nmol/kg, s.c, q.d., *n* = 15) and combination treatment with lisinopril (30 mg/kg, p.o., q.d., *n* = 14) was performed for 11 weeks in *db*/*db* UNx-ReninAAV mice. Vehicle-dosed (s.c, q.d.) *db*/*db* UNx-ReninAAV mice (*n* = 15) served as controls. ** *p* < 0.01, *** *p* < 0.001 vs. vehicle-dosed *db*/*db* UNx-ReninAAV mice. ^##^ *p* < 0.01, ^###^ *p* < 0.001 vs. semaglutide (Dunnett’s test one-factor/two-factor linear model with interaction).

Blood pressure was monitored after 1 and 10 weeks of treatment. Compared to vehicle controls, semaglutide significantly reduced hypertension in *db*/*db* UNx-ReninAAV mice. After 1 week of treatment, semaglutide reduced both diastolic (104 ± 8 mm Hg vs. 148 ± 9 mm Hg, *p* < 0.001, Figure 3A) and systolic blood pressure (134 ± 8 mm Hg vs. 178 ± 10 mm Hg, *p* < 0.01, Figure 3B). The antihypertensive effect of semaglutide was sustained over the entire treatment period, as indicated by similar nominal reductions in diastolic (102 ± 8 mm Hg vs. 144 ± 7 mm Hg, *p* < 0.001) and systolic blood pressure (135 ± 8 mm Hg vs. 181 ± 8 mm Hg, *p* < 0.001) after 10 weeks of treatment (Figure 3A,B). Correspondingly, similar reductions in mean arterial blood pressure were observed (Figure 3C). When combined with lisinopril administration, this led to further improvements in diastolic (week 1, 64 ± 5 mm Hg, *p* < 0.001 vs. vehicle; *p* < 0.01 vs. semaglutide; week 10, 68 ± 4 mm Hg, *p* < 0.001 vs. vehicle; *p* < 0.01 vs. semaglutide), systolic (week 1, 84 ± 6 mm Hg, *p* < 0.001 vs. vehicle, *p* < 0.001 vs. semaglutide; week 10, 90 ± 5 mm Hg, *p* < 0.001 vs. vehicle, *p* < 0.001 vs. semaglutide) and mean arterial blood pressure (week 1, 71 ± 5 mm Hg, *p* < 0.001 vs. vehicle, *p* < 0.001 vs. semaglutide; week 10, 76 ± 5 mm Hg, *p* < 0.001 vs. vehicle, *p* < 0.01 vs. semaglutide).

### 3.3. Semaglutide Improves Urinary Biomarkers in db/db UNx-ReninAAV Mice

**Figure 4:**
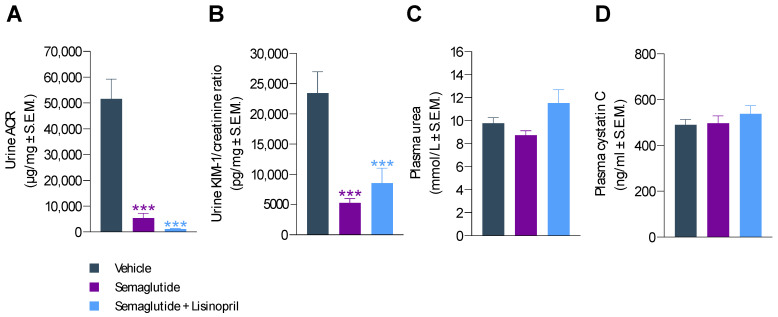
Semaglutide improves urinary markers in *db*/*db* UNx-ReninAAV mice. (**A**) Urine albumin-to-creatine ratio (ACR). (**B**) Urine kidney molecule-1 (KIM-1)-to-creatine ratio. (**C**) Plasma urea concentrations. (**D**) Plasma cystatin C levels. Semaglutide monotherapy (30 nmol/kg, s.c, q.d., *n* = 15) and combination treatment with lisinopril (30 mg/kg, p.o., q.d., *n* = 14) was performed for 11 weeks in *db*/*db* UNx-ReninAAV mice. Vehicle-dosed (s.c, q.d.) *db*/*db* UNx-ReninAAV mice (*n* = 15) served as controls. *** *p* < 0.001 vs. vehicle-dosed *db*/*db* UNx-ReninAAV mice (Dunnett’s test one-factor linear model with interaction).

Semaglutide treatment for 11 weeks substantially improved urinary biomarkers of renal function in *db*/*db* UNx-ReninAAV mice. Compared to vehicle controls, severe albuminuria was markedly reduced by semaglutide (albumin-to-creatine ratio (ACR); 5417 ± 1825 vs. 51,573 ± 7669 µg/mg, *p* < 0.001), see Figure 4A and Figure S1. Correspondingly, KIM-1-to-creatine ratio was also markedly lowered by semaglutide treatment (5223 ± 738 vs. 23,474 ± 3517 µg/mg, *p* < 0.001, Figure 4A and Figure S1). While co-administration of lisinopril further reduced ACR (1143 ± 188 µg/mg, *p* < 0.001 vs. vehicle control, Figure 4A), this effect was not significantly greater compared to semaglutide alone (*p* = 0.536). Semaglutide + lisinopril reduced KIM-1-to-creatine ratio (8540 ± 2487 µg/mg, *p* < 0.001 vs. vehicle control) to a similar extent as semaglutide monotherapy (Figure 4B). Plasma urea and cystatin C levels were unaffected by treatments (Figure 4C,D).

### 3.4. Semaglutide Improves Kidney Histopathology in db/db UNx-ReninAAV Mice

**Figure 5:**
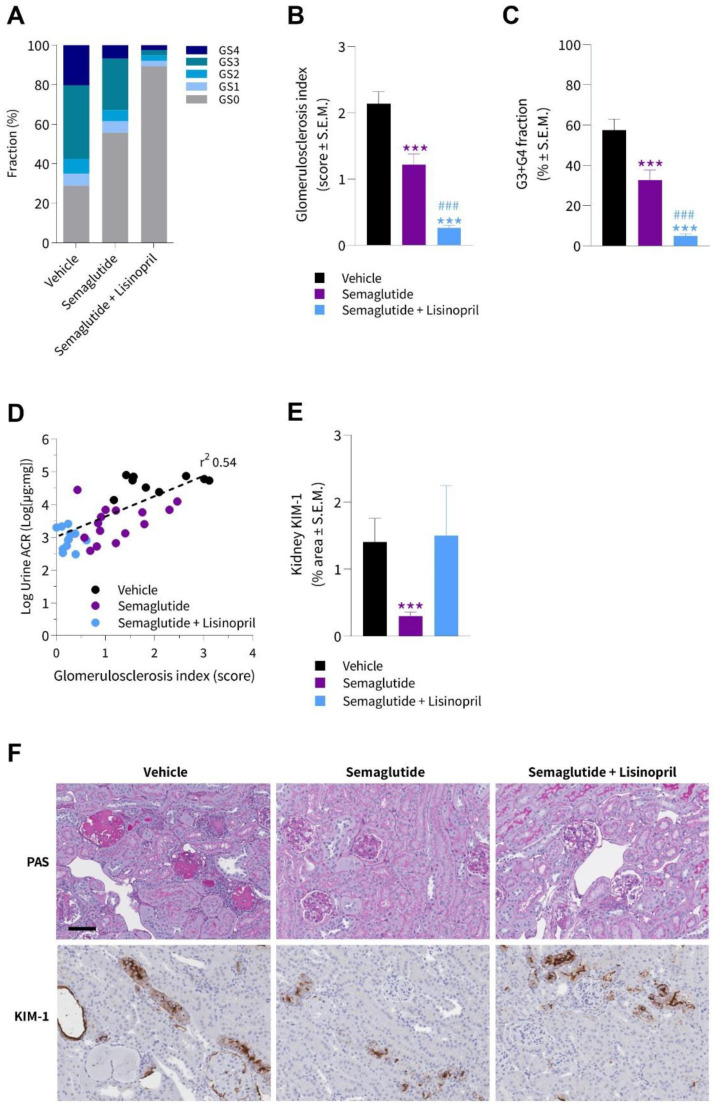
Semaglutide improves glomerulosclerosis in *db*/*db* UNx-ReninAAV mice. Automated AI-based detection of glomerulosclerosis in PAS-positive glomeruli in *db*/*db* UNx-ReninAAV mice receiving (q.d.) vehicle, semaglutide (30 nmol/kg, s.c., *n* = 15) or semaglutide (30 nmol/kg, s.c., *n* = 15) + lisinopril (30 mg/kg, p.o., *n* = 14). (**A**) Group-wise distribution (fraction %) of glomerulosclerosis scores. (**B**) Glomerulosclerosis index. (**C**) Fraction of glomeruli with severe or global glomerulosclerosis (GS3 + GS4). (**D**) Correlation of glomerulosclerosis index and albuminuria. (**E**) Fractional (%) area of whole-section kidney molecule-1 (KIM-1). (**F**) Representative photomicrographs from *db*/*db* UNx-ReninAAV mice receiving vehicle, semaglutide or combined semaglutide + lisinopril administration. *** *p* < 0.001 vs. vehicle-dosed *db*/*db* UNx-ReninAAV mice; ^###^ *p* < 0.001 vs. semaglutide (Dunnett’s test one-factor linear model with interaction). Scale bar, 100 µm.

A deep-learning-based imaging method was applied for automated detection of glomeruli and scoring of glomerulosclerosis [14]. Glomeruli were classified according to a five-point scale, i.e., GS0 (normal), GS1 (mild, sclerotic area up to 25%), GS2 (moderate, sclerotic area of 25–50%), GS3 (severe, sclerotic area of 51–75%), and GS4 (global, sclerotic area of 76–100%). Groupwise distribution of glomerulosclerosis scores is shown in Figure 5A. Vehicle-dosed animals displayed a substantial fraction of glomeruli with severe (G3) or global (G4) glomerulosclerosis. Notably, semaglutide reduced the overall degree of glomerular injury, expressed as significantly lowered glomerulosclerosis index (1.2 ± 0.2 vs. 2.1 ± 0.2, *p* < 0.001, Figure 5A) and fraction of glomeruli exhibiting severe or global sclerotic damage (GS3 + GS4, 32 ± 5 vs. 57 ± 6, *p* < 0.001, Figure 5C). The glomeruloprotective effect was even more pronounced when semaglutide was combined with lisinopril (Figure 5B). As for semaglutide alone, the improvements were particularly driven by a reduction in the fraction of glomeruli with severe or global glomerulosclerosis score following semaglutide + lisinopril treatment (Figure 5C). Lowered glomerulosclerosis index significantly correlated with reduced albuminuria (r^2^ = 0.54, *p* < 0.001, Figure 5D). Quantitative histological analysis of KIM-1 positive staining showed significantly decreased KIM-1 proportional area in the semaglutide-treated group compared to vehicle controls (0.3 ± 0.1 vs. 1.4 ± 0.4%, *p* < 0.001), while no significant difference was observed with combined semaglutide + lisinopril treatment (1.5 ± 0.7, *p* > 0.05), see Figure 5E. Representative images of glomerulosclerosis pathology visualized with a PAS staining and KIM-1 immunohistochemistry is shown in Figure 5F.

### 3.5. Semaglutide Improves Podocyte Health in db/db UNx-ReninAAV Mice

**Figure 6:**
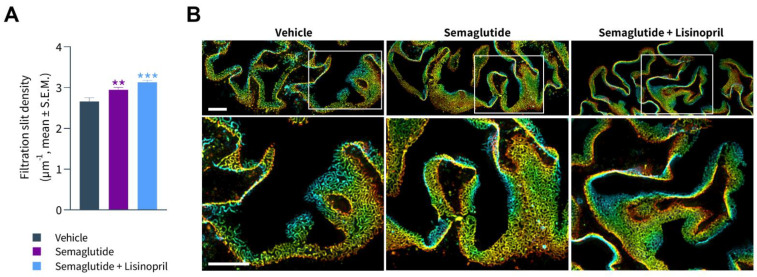
Semaglutide improves podocyte foot process ultrastructure in *db*/*db* UNx-ReninAAV mice. (**A**) Semaglutide monotherapy (30 nmol/kg, s.c., q.d. *n* = 15) and combined lisinopril treatment (30 mg/kg, p.o., *n* = 14) reduces severity of podocyte foot process effacement in *db*/*db* UNx-ReninAAV mice, as indicated by increased filtration slit density (FSD). Vehicle-dosed (s.c, q.d.) *db*/*db* UNx-ReninAAV mice (*n* = 15) served as controls. ** *p* < 0.01, *** *p* < 0.001 vs. vehicle-dosed *db*/*db* UNx-ReninAAV mice (one-way ANOVA). The FSD of 20 glomeruli was determined for every animal. (**B**) Upper panels: Photomicrographs of podocin-stained glomeruli obtained by wide field microscopy and after SIM reconstruction. The 3D-SIM (z-stack) images of slit diaphragms were colorized according to their position on the *z*-axis (µm). Lower panels: As shown by increased magnification, the space between podocin-stained capillary loops was increased in vehicle-dosed *db*/*db* UNx-ReninAAV mice, indicative of aberrant foot process architecture with reduced FSD. Compared to vehicle controls, semaglutide and semaglutide + lisinopril significantly increased FSD, hence improving podocyte health in *db*/*db* UNx-ReninAAV mice. Scale bars, 5 µm.

FSD was assessed using synaptopodin (a foot process actin-associated protein) and podocin (a raft-associated component of the glomerular slit diaphragm) as markers of phenotypic maturity of podocytes. Rarefication of foot processes was evident in *db*/*db* UNx-ReninAAV mice (Figure 6A). Compared to vehicle controls, effacement of foot processes was less prominent in semaglutide-treated *db*/*db* UNx-ReninAAV mice. Accordingly, semaglutide significantly increased FSD in *db*/*db* UNx-ReninAAV mice (*p* < 0.01 vs. vehicle control, Figure 6A,B). *db*/*db* UNx-ReninAAV mice receiving combined semaglutide and lisinopril treatment predominantly demonstrated perfectly formed foot processes and some areas with low effacement and, rarely, high effacement. In accordance, semaglutide + lisinopril further increased FSD compared to vehicle controls (*p* < 0.001, Figure 6A,B), however, this was not statistically significant compared to semaglutide alone (*p* = 0.08).

### 3.6. Semaglutide Promotes Discrete Renal Gene Expression Changes in db/db UNx-ReninAAV Mice

**Figure 7:**
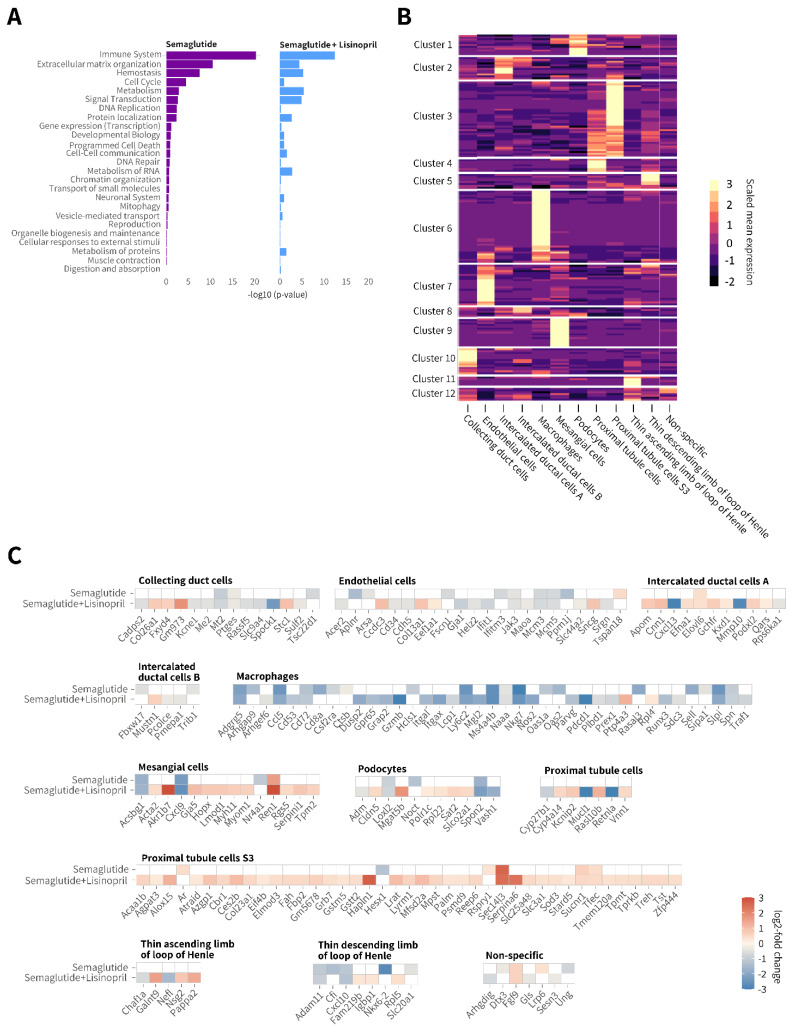
Semaglutide suppresses gene expression markers of extracellular matrix remodelling and immune system activation in *db*/*db* UNx-ReninAAV mice. (**A**) Enriched Reactome pathways following semaglutide (30 nmol/kg, s.c., q.d. *n* = 7) and semaglutide (30 nmol/kg, s.c., q.d.) + lisinopril (30 mg/kg, p.o., *n* = 7) treatment compared to corresponding vehicle-dosed *db*/*db* UNx-ReninAAV mice (*n* = 7). (**B**) Deconvolution of differentially expressed genes (DEGs) according to major kidney cell type. Bright yellow color in heatmap denotes cluster of genes expressed with relative high cell specificity. (**C**) Heatmaps illustrating expression changes in individual DEGs grouped according to major cell type expressing the corresponding gene. Data are expressed relative to vehicle-dosed controls. Color gradients indicate significantly upregulated (red color) or downregulated (blue color) gene expression (log2-fold change, false discovery rate (FDR) < 0.05). All DEGs are listed in Table S1. Bulk RNA sequencing data are accessible using a web-based global gene expression data view-er [Gubra Gene Expression Experience (GGEX), https://rnaseq.gubra.dk/, accessed on 3 July 2022].

Semaglutide promoted highly discrete renal gene expression changes in *db*/*db* UNx-ReninAAV mice (*n* = 57 differentially expressed genes (DEGs) vs. vehicle control). Co-administration of lisinopril resulted in relatively more widespread changes in gene expression (*n* = 158 DEGs vs. vehicle control), showing partial overlap (*n* = 27 DEGs) with semaglutide monotherapy (Table S1). Enriched Reactome pathways suggested the largest proportion of DEGs were particularly associated with the immune system and extracellular matrix (ECM) remodeling (Figure 7A). Bulk RNA sequencing data are accessible using a web-based global gene expression data viewer [Gubra Gene Expression Experience (GGEX), https://rnaseq.gubra.dk/, accessed on 3 July 2022]. This open access resource can be used for further data mining on global gene expression changes. Cell type deconvolution was based on single-nucleus RNA sequencing data previously reported in the *db*/*db* UNx-ReninAAV mouse [15]. Individual DEGs obtained from bulk RNA sequencing analysis were assigned to kidney cell types predominantly expressing the corresponding regulated gene (Figure 7B, individual DEGs are listed in Table S1). DEGs were linked to 11 specific kidney cell types, including proximal tubule cells (*n* = 41 DEGs), macrophages (*n* = 39 DEGs), endothelial cells (*n* = 22 DEGs), intercalated ductal cells (*n* = 16 DEGs), mesangial cells (*n* = 14 DEGs), collecting duct cells (*n* = 14 DEGs), thin ascending/descending loop of Henle cells (*n* = 13 DEGs), and podocytes (*n* = 11 DEGs). Cell type assignment and directional change in individual DEGs (relative to vehicle controls) is illustrated in Figure 7C. Suppression of macrophage-associated genes was the most notable kidney transcriptional signature following semaglutide monotherapy. In general, perturbations in renal gene expression were more extensive following semaglutide + lisinopril co-treatment. Bulk RNA sequencing indicated low *Glp1r* expression in kidneys of *db*/*db* UNx-ReninAAV mice (data not shown).

## 4. Discussion

The present study is the first to demonstrate nephroprotective effects of semaglutide in a preclinical model of advanced DKD. We here demonstrate that semaglutide robustly improves renal outcomes in the *db*/*db* UNx-ReninAAV mouse model of hypertension-accelerated DKD. Notably, semaglutide reduced glomerulosclerosis severity concurrent with substantial improvements in hyperglycemia, hypertension and albuminuria. Further improvements in kidney pathology were achieved by adjunctive standard of care using an ACE inhibitor (lisinopril). Collectively, the current study supports the development of GLP-1R agonists for management of DKD.

Recent clinical trials have demonstrated beneficial effects GLP-1R agonists on renal outcomes, especially in patients with T2D who are at high risk for developing cardiovascular disease [9,28,29]. To date, clinical trials with GLP-1 receptor agonists have not included kidney events as primary endpoint. The ongoing FLOW semaglutide trial (clinicaltrials.gov, #NCT03819153) is therefore the first to specifically addresses whether a long-acting GLP-1R agonist can slow the progression of DKD. In addition to hyperglycemia, obesity and hypertension are considered essential factors in the development and progression of DKD [1,30]. The mode of action underlying the therapeutic effects of GLP-1R agonists in DKD is incompletely understood and likely determined by both extra- and intrarenal action [30]. The present study therefore aimed to profile long-term semaglutide treatment in a preclinical model with closer resemblance to human DKD pathogenesis. To this end, we characterized semaglutide monotreatment with and without combined antihypertensive standard of care in *db*/*db* UNx-ReninAAV mice, a preclinical model of hypertension-accelerated DKD [13,14,31].

In line with previous rodent studies [32,33], semaglutide promoted sustained body weight loss and profound improvement in glycemic control in diabetic *db*/*db* UNx-ReninAAV mice. Co-administration of lisinopril resulted in slightly less weight loss. It is well established that *db*/*db* mice exhibit a gradual decline in growth rate due to worsening of glucosuria resulting in progressive loss of calories [34,35]. ACE inhibitors can enhance whole-body and skeletal muscle glucose disposal, presumably by increasing insulin receptor signaling and GLUT-4 glucose transporter function [36]. Given that lisinopril is weight-neutral in *db*/*db* UNx-ReninAAV mice [14], it is possible that semaglutide and lisinopril combination treatment may have synergistic effects on insulin sensitivity to improve overall metabolic status in the model. It is noteworthy that semaglutide also improved hypertension in *db*/*db* UNx-ReninAAV, being consistent with studies in T2D patients [9,10]. Concurrent with robust reductions in systolic and diastolic blood pressure, semaglutide treatment led to marked improvements in albuminuria and glomerulosclerosis. Other antidiabetic drug classes have previously been profiled in *db*/*db* UNx-ReninAAV mice, including rosiglitazone (peroxisome proliferator-activated receptor-γ agonist) and empagliflozin (SGLT2 inhibitor), which robustly reduces hyperglycemia, without, however, influencing hypertension and kidney histopathology in the model [13,14,31]. Semaglutide may therefore likely improve kidney outcomes in *db*/*db* UNx-ReninAAV mice regardless of the robust antihyperglycemic effect. Previous studies have indicated that separate treatment of hypertension with lisinopril partially reverses albuminuria and glomerulosclerosis in *db*/*db* UNx-ReninAAV mice [13,14,31]. Notably, semaglutide and lisinopril combination treatment exerted even more robust effects on renal histological outcomes. We therefore speculate that nephroprotective activity of semaglutide could, at least in part, be linked to antihypertensive action.

It is unresolved if GLP-1R agonists can influence the activity of the RAAS system. While GLP-1R agonists have been demonstrated to attenuate hypertension in response to high-salt load and angiotensin-II infusion in rodents [37,38], human studies suggest that GLP-1 receptor agonists can reduce circulating angiotensin-II levels in the absence of blood pressure effects [39,40,41]. GLP-1R agonist effects on plasma renin levels are also inconclusive, being either unchanged [39,40,41,42] or decreased [43]. In line with a recent report demonstrating liraglutide and semaglutide-stimulated renin mRNA expression in mouse renal vasculature in vivo [44], semaglutide with and without lisinopril co-administration significantly increased kidney renin gene expression *db*/*db* UNx-ReninAAV mice. It should be noted that kidney pathology in *db*/*db* UNx-ReninAAV mice is considered independent of intrarenal renin expression, as renin overexpression is driven by a liver-specific promoter whereupon the gene product is released into the circulation [13,31], likely explaining the compensatory downregulation of renal renin gene expression in the model [14]. While the implications of GLP-1R agonist-stimulated intrarenal renin expression in mice are unclear, RAAS-independent function of renin-expressing cells has been reported to be involved in glomerular regenerative processes [45]. The antihypertensive action of GLP-1R agonists may be secondary to the well-described diuretic and natriuretic effects of this drug class, pointing to the possibility that GLP-1R agonists afford nephroprotection by improving renal hemodynamics [38,40,46,47,48]. Increased glomerular capillary blood pressure, resulting in hemodynamic injury to the glomerular cells, is considered a critical pathophysiological factor in the onset and progression of DKD [49]. In support of intrarenal hemodynamic effects of GLP-1R agonists, localization of GLP-1Rs in the kidney is reported to be restricted to vascular smooth muscle cells [47,48,50], and GLP-1R activation is suggested to mediate renal vasorelaxant and blood flow stimulation to preserve glomerular function [47,48,51].

Damage to the glomerular filtration barrier (GFB), consisting of a fenestrated endothelium, the glomerular basement membrane, and a layer of specialized interdigitating epithelial cells, termed podocytes, plays a fundamental role in progressive development of proteinuria and glomerulosclerosis in DKD [52]. *db*/*db* UNx-ReninAAV mice demonstrated podocyte foot effacement, predicting disruption of the slit diaphragm connecting podocyte foot processes [53]. The changes in podocyte ultrastructure were observed in the context of massive albuminuria in *db*/*db* UNx-ReninAAV mice. It is therefore noteworthy that the modest, however significant, increase in FSD in *db*/*db* UNx-ReninAAV mice receiving semaglutide as monotherapy or combined with lisinopril treatment was paralleled by a marked attenuation of albuminuria. Mounting evidence suggests that filtration slits, established by the interdigitations of podocytes, may be the ultimate and more selective barrier for the majority of proteins [54,55]. Accordingly, retraction and effacement of podocyte tertiary foot processes, reducing the podocyte surface area and disrupting the slit diaphragm, represents one of the earliest structural features of impaired GFB function [56]. Furthermore, recent ultrastructural analyses indicate that changes in the density and morphology of podocyte foot processes leads to capillary dilation as result of reduced compressive forces serving to counteract filtration pressure, leading to deficient GFB function and progressive proteinuria [57]. Consequently, the function of podocytes is largely based on the cell architecture, and structural injury and loss of podocytes is strongly correlated with albuminuria [58]. In agreement, even small changes in FSD have been demonstrated to have profound effects on the ACR [57]. Therefore, the modest absolute increases in FSD following long-term semaglutide administration with and without lisinopril co-treatment could, at least in part, explain near-normalization of ACR in *db*/*db* UNx-ReninAAV mice. Hence, protective effects of semaglutide on podocyte morphology may contribute to improve severe proteinuria and advanced glomerulosclerosis in *db*/*db* UNx-ReninAAV mice.

Although it has been reported that native GLP-1 prevents glucotoxicity in cultured murine podocytes by suppressing release of pro-inflammatory cytokines [59], GLP-1R expression in podocytes has so far not been established. It should therefore be considered that benefits on podocyte health may be indirect and potentially relate to anti-inflammatory effects of GLP-1R agonists [30,60,61,62]. In support of this notion, semaglutide downregulated a considerable number of macrophage-associated genes in *db*/*db* UNx-ReninAAV mice, suggesting reduced macrophage infiltration and activation. Inhibition of macrophage-driven inflammation is further substantiated by the observation that semaglutide reduced plasma and renal KIM-1 levels, a potential marker of macrophage-driven tubule interstitial inflammation [63]. Compared to vehicle controls, semaglutide promoted considerably greater relative reductions in plasma KIM-1 levels than observed for kidney KIM-1 expression. Hence, changes in KIM-1 secretion may represent a more sensitive and reliable marker of kidney health. This may also explain why combined treatment with semaglutide and lisinopril promoted highly robust reductions in circulating KIM-1 levels without changing kidney KIM-1 expression. It has previously been established that perturbations in immune system and extracellular matrix remodeling signaling pathways are essential disease drivers in *db*/*db* UNx-ReninAAV mice [13,15]. In line with anti-inflammatory effects of semaglutide in this model, liraglutide has been reported to improve renal outcomes by decreasing renal immune cell infiltration and inflammation in a non-diabetic nephrotoxic serum nephritis mouse model of CKD [64,65].

## 5. Conclusions

Semaglutide improves disease hallmarks in the *db*/*db* UNx-ReninAAV mouse model of advanced DKD. Renal outcomes were further improved by combined antihypertensive standard of care. Collectively, our data support clinical development of semaglutide for the treatment of DKD, and further highlight the use of *db*/*db* UNx-ReninAAV mice for preclinically profiling novel compounds with potential therapeutic effect in DKD.

## Data Availability

Bulk RNA sequencing data are accessible at a web-based gene expression data viewer [Gubra Gene Expression Experience (GGEX), https://rnaseq.gubra.dk/ (accessed on 3 July 2022)]. Other data are available on reasonable request.

## Supplementary Materials

The following supporting information can be downloaded at: https://www.mdpi.com/article/10.3390/biomedicines10071661/s1, Figure S1: Terminal urine levels of creatinine, albumin and kidney injury molecule-1 (KIM-1) in db/db UNx-ReninAAV mice; Table S1: Deconvolution of differentially expressed genes according to kidney cell type.
