# Supplementary material for: Nephroprotective Effects of Semaglutide as Mono- and Combination Treatment with Lisinopril in a Mouse Model of Hypertension-Accelerated Diabetic Kidney Disease
Source: Biomedicines. 2022 Jul 11;10(7):1661. doi: 10.3390/biomedicines10071661 (PMC9313388; doi:10.3390/biomedicines10071661)
Supplement: Supplementary file 1 [file biomedicines-10-01661-s001.zip › biomedicines-1785974-supplementary.pdf]

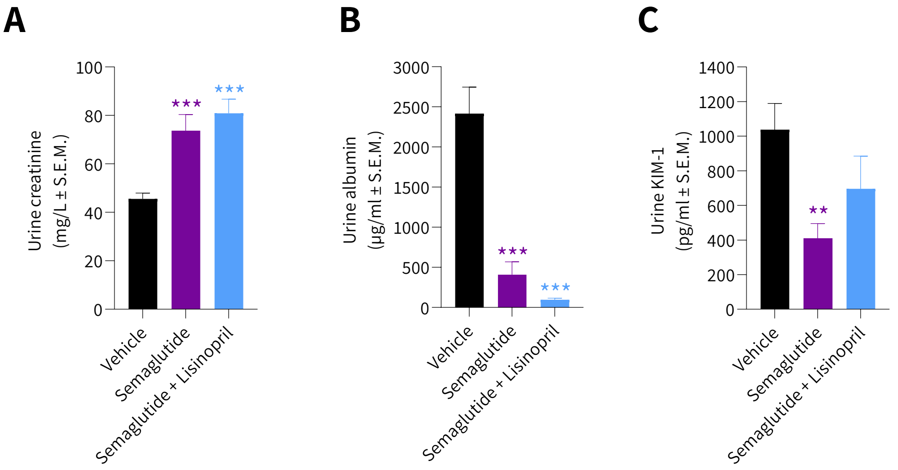

Supplementary Figure S1. Terminal urine levels of creatinine, albumin and kidney injury molecule-1 (KIM-1) in *db/db* UNx-ReninAAV mice treated (QD) with vehicle (SC), semaglutide (30 nmol/kg, SC), or semaglutide (30 nmol/kg, SC) + lisinopril (30 mg/kg, PO) for 11 weeks.

Cluster	Predominant cell type	Gene name	Description	Sema	Sema+Lisi	Major Reactome pathway
1	Podocytes	Adm	adrenomedullin	Not regulated	Down-regulated	Signal Transduction
1	Podocytes	Cldn5	claudin 5	Not regulated	Upregulated	
1	Podocytes	Loxl2	lysyl oxidase-like 2	Down-regulated	Down-regulated	Extracellular matrix organization
1	Podocytes	Mgat5b	mannoside acetylglucosaminyltransferase 5, isoenzyme B	Not regulated	Upregulated	
1	Podocytes	Noct	nocturnin	Down-regulated	Not regulated	
1	Podocytes	Polr1c	polymerase (RNA) I polypeptide C	Not regulated	Upregulated	Epigenetic regulation of gene expression
1	Podocytes	Rpl22	ribosomal protein L22	Not regulated	Upregulated	
1	Podocytes	Sat2	spermidine/spermine N1-acetyl transferase 2	Not regulated	Upregulated	
1	Podocytes	Slco2a1	solute carrier organic anion transporter family, member 2a1	Not regulated	Upregulated	Transport of small molecules
1	Podocytes	Spon2	spondin 2, extracellular matrix protein	Down-regulated	Down-regulated	Metabolism of proteins
1	Podocytes	Vash1	vasohibin 1	Not regulated	Down-regulated	
2	Intercalated ductal cells A	AA467197	expressed sequence AA467197	Not regulated	Down-regulated	
2	Intercalated ductal cells A	Apom	apolipoprotein M	Not regulated	Upregulated	Metabolism|Signal Transduction
2	Intercalated ductal cells A	Cnn1	calponin 1	Not regulated	Upregulated	
2	Intercalated ductal cells A	Cxcl13	chemokine (C-X-C motif) ligand 13	Not regulated	Down-regulated	Signal Transduction
2	Intercalated ductal cells A	Efna1	ephrin A1	Not regulated	Upregulated	Developmental Biology
2	Intercalated ductal cells A	Elovl6	ELOVL family member 6, elongation of long chain fatty acids (yeast)	Upregulated	Upregulated	Metabolism
2	Intercalated ductal cells A	Gchfr	GTP cyclohydrolase I feedback regulator	Not regulated	Upregulated	Metabolism
2	Intercalated ductal cells A	Kxd1	KxDL motif containing 1	Not regulated	Upregulated	
2	Intercalated ductal cells A	Mmp10	matrix metallopeptidase 10	Not regulated	Down-regulated	Collagen degradation
2	Intercalated ductal cells A	Podxl2	podocalyxin-like 2	Not regulated	Upregulated	Metabolism
2	Intercalated ductal cells A	Qars	glutaminyl-tRNA synthetase	Not regulated	Upregulated	
2	Intercalated ductal cells A	Rps6ka1	ribosomal protein S6 kinase polypeptide 1	Not regulated	Down-regulated	Neurotransmitter receptors and postsynaptic signal transmission
3	Proximal tubule cells S3	Acaa1b	acetyl-Coenzyme A acyltransferase 1B	Not regulated	Upregulated	Metabolism
3	Proximal tubule cells S3	Agpat3	1-acylglycerol-3-phosphate O-acyltransferase 3	Not regulated	Upregulated	Metabolism
3	Proximal tubule cells S3	Alox15	arachidonate 15-lipoxygenase	Not regulated	Upregulated	Metabolism
3	Proximal tubule cells S3	Ar	androgen receptor	Upregulated	Not regulated	Signal Transduction
3	Proximal tubule cells S3	Atraid	all-trans retinoic acid induced differentiation factor	Not regulated	Upregulated	
3	Proximal tubule cells S3	Azgp1	alpha-2-glycoprotein 1, zinc	Not regulated	Upregulated	Transport of small molecules
3	Proximal tubule cells S3	Cbr1	carbonyl reductase 1	Not regulated	Upregulated	Metabolism
3	Proximal tubule cells S3	Ces2b	carboxyesterase 2B	Not regulated	Upregulated	Metabolism
3	Proximal tubule cells S3	Col23a1	collagen, type XXIII, alpha 1	Not regulated	Upregulated	Extracellular matrix organization
3	Proximal tubule cells S3	Eif4b	eukaryotic translation initiation factor 4B	Not regulated	Upregulated	L13a-mediated translational silencing of Ceruloplasmin expression
3	Proximal tubule cells S3	Elmod3	ELMO/CED-12 domain containing 3	Not regulated	Upregulated	
3	Proximal tubule cells S3	Fah	fumarylacetoacetate hydrolase	Not regulated	Upregulated	Metabolism
3	Proximal tubule cells S3	Fbp2	fructose bisphosphatase 2	Not regulated	Upregulated	Metabolism
3	Proximal tubule cells S3	Gm5678	predicted gene 5678	Not regulated	Upregulated	Metabolism
3	Proximal tubule cells S3	Grb7	growth factor receptor bound protein 7	Not regulated	Upregulated	Hemostasis
3	Proximal tubule cells S3	Gstm5	glutathione S-transferase, mu 5	Not regulated	Upregulated	Metabolism
3	Proximal tubule cells S3	Gstt2	glutathione S-transferase, theta 2	Not regulated	Upregulated	Metabolism
3	Proximal tubule cells S3	Hapln1	hyaluronan and proteoglycan link protein 1	Not regulated	Upregulated	Extracellular matrix organization
3	Proximal tubule cells S3	Hesx1	homeobox gene expressed in ES cells	Down-regulated	Not regulated	
3	Proximal tubule cells S3	Lrat	lecithin-retinol acyltransferase (phosphatidylcholine-retinol-O-acyltransferase)	Not regulated	Upregulated	Metabolism|Signal Transduction
3	Proximal tubule cells S3	Lyrm1	LYR motif containing 1	Not regulated	Upregulated	
3	Proximal tubule cells S3	Mfsd2a	major facilitator superfamily domain containing 2A	Not regulated	Upregulated	Metabolism
3	Proximal tubule cells S3	Mpst	mercaptopyruvate sulfurtransferase	Not regulated	Upregulated	Metabolism
3	Proximal tubule cells S3	Palm	paralemmin	Not regulated	Upregulated	
3	Proximal tubule cells S3	Psmd9	proteasome (prosome, macropain) 26S subunit, non-ATPase, 9	Not regulated	Upregulated	Cell Cycle
3	Proximal tubule cells S3	Reep6	receptor accessory protein 6	Not regulated	Upregulated	Signal Transduction
3	Proximal tubule cells S3	Rspry1	ring finger and SPRY domain containing 1	Upregulated	Not regulated	
3	Proximal tubule cells S3	Sec14l3	SEC14-like lipid binding 3	Upregulated	Upregulated	
3	Proximal tubule cells S3	Serpina6	serine (or cysteine) peptidase inhibitor, clade A, member 6	Not regulated	Upregulated	
3	Proximal tubule cells S3	Slc25a48	solute carrier family 25, member 48	Not regulated	Upregulated	
3	Proximal tubule cells S3	Slc3a1	solute carrier family 3, member 1	Not regulated	Upregulated	Amino acid transport across the plasma membrane
3	Proximal tubule cells S3	Sod3	superoxide dismutase 3, extracellular	Not regulated	Upregulated	Cellular responses to stress
3	Proximal tubule cells S3	Stard5	StAR-related lipid transfer (START) domain containing 5	Not regulated	Upregulated	Metabolism
3	Proximal tubule cells S3	Sucnr1	succinate receptor 1	Upregulated	Upregulated	Signal Transduction
3	Proximal tubule cells S3	Tfec	transcription factor EC	Upregulated	Upregulated	
3	Proximal tubule cells S3	Tmem120a	transmembrane protein 120A	Not regulated	Upregulated	
3	Proximal tubule cells S3	Tpmt	thiopurine methyltransferase	Not regulated	Upregulated	Metabolism
3	Proximal tubule cells S3	Tprkb	Tp53rk binding protein	Not regulated	Upregulated	
3	Proximal tubule cells S3	Treh	trehalase (brush-border membrane glycoprotein)	Not regulated	Upregulated	
3	Proximal tubule cells S3	Tst	thiosulfate sulfurtransferase, mitochondrial	Not regulated	Upregulated	Metabolism
3	Proximal tubule cells S3	Zfp444	zinc finger protein 444	Not regulated	Upregulated	
4	Proximal tubule cells	Cyp27b1	cytochrome P450, family 27, subfamily b, polypeptide 1	Not regulated	Down-regulated	Metabolism
4	Proximal tubule cells	Cyp4a14	cytochrome P450, family 4, subfamily a, polypeptide 14	Not regulated	Upregulated	Metabolism
4	Proximal tubule cells	Kcnip2	Kv channel-interacting protein 2	Not regulated	Upregulated	Muscle contraction
4	Proximal tubule cells	Mucl1	mucin-like 1	Not regulated	Down-regulated	
4	Proximal tubule cells	Rasl10b	RAS-like, family 10, member B	Not regulated	Upregulated	
4	Proximal tubule cells	Retnla	resistin like alpha	Not regulated	Down-regulated	
4	Proximal tubule cells	Vnn1	vanin 1	Not regulated	Upregulated	Metabolism
5	Thin descending limb of of loop of Henle	Adam11	a disintegrin and metallopeptidase domain 11	Down-regulated	Down-regulated	Developmental Biology
5	Thin descending limb of of loop of Henle	Cfi	complement component factor i	Down-regulated	Not regulated	Complement cascade
5	Thin descending limb of of loop of Henle	Cxcl10	chemokine (C-X-C motif) ligand 10	Down-regulated	Down-regulated	Signal Transduction
5	Thin descending limb of of loop of Henle	Fam219b	family with sequence similarity 219, member B	Not regulated	Upregulated	
5	Thin descending limb of of loop of Henle	Igbp1	immunoglobulin (CD79A) binding protein 1	Not regulated	Upregulated	
5	Thin descending limb of of loop of Henle	Nkx6-2	NK6 homeobox 2	Down-regulated	Not regulated	
5	Thin descending limb of of loop of Henle	Rpl5	ribosomal protein L5	Not regulated	Upregulated	
5	Thin descending limb of of loop of Henle	Slc20a1	solute carrier family 20, member 1	Down-regulated	Not regulated	Transport of small molecules
6	Macrophages	Adgrg5	adhesion G protein-coupled receptor G5 (GPR114)	Down-regulated	Down-regulated	
6	Macrophages	Arhgap9	Rho GTPase activating protein 9	Down-regulated	Not regulated	Signal Transduction
6	Macrophages	Arhgef6	Rac/Cdc42 guanine nucleotide exchange factor (GEF) 6	Not regulated	Down-regulated	Cell-Cell communication
6	Macrophages	Ccl5	chemokine (C-C motif) ligand 5	Down-regulated	Down-regulated	Signal Transduction
6	Macrophages	Cd53	CD53 antigen	Not regulated	Down-regulated	Innate Immune System
6	Macrophages	Cd72	CD72 antigen	Down-regulated	Down-regulated	Developmental Biology

6	Macrophages	Cd8a	CD8 antigen, alpha chain	Down-regulated	Not regulated	Adaptive Immune System
6	Macrophages	Csf2ra	colony stimulating factor 2 receptor, alpha, low-affinity (granulocyte-macrophage)	Down-regulated	Not regulated	Cytokine Signaling in Immune system
6	Macrophages	Ctsb	cathepsin B	Down-regulated	Not regulated	Adaptive Immune System
6	Macrophages	Dusp2	dual specificity phosphatase 2	Not regulated	Down-regulated	RAF-independent MAPK1/3 activation
6	Macrophages	Gpr65	G-protein coupled receptor 65	Not regulated	Down-regulated	Signal Transduction
6	Macrophages	Grap2	GRB2-related adaptor protein 2	Not regulated	Down-regulated	Adaptive Immune System
6	Macrophages	Gzmb	granzyme B	Not regulated	Down-regulated	Apoptosis
6	Macrophages	Hcls1	hematopoietic cell specific Lyn substrate 1	Down-regulated	Not regulated	
6	Macrophages	Itgal	integrin alpha L	Not regulated	Down-regulated	Hemostasis
6	Macrophages	Itgax	integrin alpha X	Not regulated	Down-regulated	Hemostasis
6	Macrophages	Lcp1	lymphocyte cytosolic protein 1	Not regulated	Down-regulated	
6	Macrophages	Ly6c2	lymphocyte antigen 6 complex, locus C2	Down-regulated	Down-regulated	
6	Macrophages	Mgl2	macrophage galactose N-acetyl-galactosamine specific lectin 2	Not regulated	Down-regulated	
6	Macrophages	Ms4a4b	membrane-spanning 4-domains, subfamily A, member 4B	Down-regulated	Down-regulated	
6	Macrophages	Naaa	N-acylethanolamine acid amidase	Down-regulated	Down-regulated	Neurotransmitter release cycle
6	Macrophages	Nkg7	natural killer cell group 7 sequence	Down-regulated	Down-regulated	
6	Macrophages	Nos2	nitric oxide synthase 2, inducible	Not regulated	Down-regulated	Hemostasis
6	Macrophages	Oas1a	2'-5' oligoadenylate synthetase 1A	Down-regulated	Not regulated	
6	Macrophages	Oas2	2'-5' oligoadenylate synthetase 2	Down-regulated	Not regulated	
6	Macrophages	Parvg	parvin, gamma	Not regulated	Down-regulated	
6	Macrophages	Pdcd1	programmed cell death 1	Not regulated	Down-regulated	Adaptive Immune System
6	Macrophages	Plbd1	phospholipase B domain containing 1	Not regulated	Down-regulated	Metabolism
6	Macrophages	Prex1	phosphatidylinositol-3,4,5-trisphosphate-dependent Rac exchange factor 1	Not regulated	Down-regulated	Signal Transduction
6	Macrophages	Ptp4a3	protein tyrosine phosphatase 4a3	Not regulated	Upregulated	
6	Macrophages	Rasal3	RAS protein activator like 3	Down-regulated	Not regulated	Signal Transduction
6	Macrophages	Rpl4	ribosomal protein L4	Not regulated	Upregulated	
6	Macrophages	Runx3	runt related transcription factor 3	Not regulated	Down-regulated	Generic Transcription Pathway
6	Macrophages	Sdc3	syndecan 3	Not regulated	Down-regulated	Hemostasis
6	Macrophages	Sell	selectin, lymphocyte	Down-regulated	Not regulated	Hemostasis
6	Macrophages	Sipa1	signal-induced proliferation associated gene 1	Down-regulated	Not regulated	Adaptive Immune System
6	Macrophages	Slpi	secretory leukocyte peptidase inhibitor	Down-regulated	Down-regulated	Innate Immune System
6	Macrophages	Spn	sialophorin (leukosialin, CD43)	Down-regulated	Down-regulated	Hemostasis
6	Macrophages	Traf1	TNF receptor-associated factor 1	Not regulated	Down-regulated	Signal Transduction
7	Endothelial cells	Acer2	alkaline ceramidase 2	Not regulated	Down-regulated	Metabolism
7	Endothelial cells	Aplnr	apelin receptor	Down-regulated	Not regulated	Signal Transduction
7	Endothelial cells	Arsa	arylsulfatase A	Down-regulated	Not regulated	Metabolism
7	Endothelial cells	Ccdc3	coiled-coil domain containing 3	Not regulated	Upregulated	
7	Endothelial cells	Cd34	CD34 antigen	Not regulated	Down-regulated	Adaptive Immune System
7	Endothelial cells	Cdh5	cadherin 5	Not regulated	Down-regulated	Cell-Cell communication
7	Endothelial cells	Col13a1	collagen, type XIII, alpha 1	Not regulated	Upregulated	Collagen degradation
7	Endothelial cells	Eef1a1	eukaryotic translation elongation factor 1 alpha 1	Not regulated	Upregulated	
7	Endothelial cells	Fscn1	fascin actin-bundling protein 1 (fascin-1)	Down-regulated	Not regulated	
7	Endothelial cells	Gja1	gap junction protein, alpha 1	Not regulated	Down-regulated	Gap junction trafficking and regulation
7	Endothelial cells	Helz2	helicase with zinc finger 2, transcriptional coactivator	Not regulated	Down-regulated	Metabolism
7	Endothelial cells	Ifit1	interferon-induced protein with tetratricopeptide repeats 1	Not regulated	Down-regulated	
7	Endothelial cells	Ifitm3	interferon induced transmembrane protein 3	Down-regulated	Not regulated	Adaptive Immune System
7	Endothelial cells	Jak3	Janus kinase 3	Down-regulated	Down-regulated	Interleukin-7 signaling
7	Endothelial cells	Maoa	monoamine oxidase A	Not regulated	Down-regulated	Neurotransmitter clearance
7	Endothelial cells	Mcm3	minichromosome maintenance complex component 3	Down-regulated	Down-regulated	Cell Cycle
7	Endothelial cells	Mcm5	minichromosome maintenance complex component 5	Down-regulated	Down-regulated	Cell Cycle
7	Endothelial cells	Ppm1j	protein phosphatase 1J	Down-regulated	Not regulated	
7	Endothelial cells	Slc44a2	solute carrier family 44, member 2	Not regulated	Down-regulated	Metabolism
7	Endothelial cells	Sncg	synuclein, gamma	Not regulated	Upregulated	
7	Endothelial cells	Srgn	serglycin	Not regulated	Down-regulated	Hemostasis
7	Endothelial cells	Tspan18	tetraspanin 18	Upregulated	Not regulated	
8	Intercalated ductal cells B	Fbxw17	F-box and WD-40 domain protein 17	Down-regulated	Not regulated	
8	Intercalated ductal cells B	Mustn1	musculoskeletal, embryonic nuclear protein 1	Not regulated	Upregulated	
8	Intercalated ductal cells B	Pcolce	procollagen C-endopeptidase enhancer protein	Not regulated	Down-regulated	Extracellular matrix organization
8	Intercalated ductal cells B	Pmepa1	prostate transmembrane protein, androgen induced 1	Not regulated	Down-regulated	Signal Transduction
8	Intercalated ductal cells B	Trib1	tribbles pseudokinase 1	Down-regulated	Not regulated	
9	Mesangial cells	Acsbg1	acyl-CoA synthetase bubblegum family member 1	Down-regulated	Down-regulated	Metabolism
9	Mesangial cells	Acta2	actin, alpha 2, smooth muscle, aorta	Not regulated	Upregulated	Muscle contraction
9	Mesangial cells	Akr1b7	aldo-keto reductase family 1, member B7	Not regulated	Upregulated	Metabolism
9	Mesangial cells	Cxcl9	chemokine (C-X-C motif) ligand 9	Down-regulated	Down-regulated	Signal Transduction
9	Mesangial cells	Gja5	gap junction protein, alpha 5	Not regulated	Upregulated	Gap junction trafficking and regulation
9	Mesangial cells	Hopx	HOP homeobox	Not regulated	Upregulated	
9	Mesangial cells	Lmod1	leiomodin 1 (smooth muscle)	Not regulated	Upregulated	Muscle contraction
9	Mesangial cells	Myh11	myosin, heavy polypeptide 11, smooth muscle	Not regulated	Upregulated	Signal Transduction
9	Mesangial cells	Myom1	myomesin 1	Not regulated	Upregulated	
9	Mesangial cells	Nr4a1	nuclear receptor subfamily 4, group A, member 1	Down-regulated	Not regulated	PIP3 activates AKT signaling
9	Mesangial cells	Ren1	renin 1 structural	Upregulated	Upregulated	Metabolism of Angiotensinogen to Angiotensins
9	Mesangial cells	Rgs5	regulator of G-protein signaling 5	Not regulated	Upregulated	Signal Transduction
9	Mesangial cells	Serpini1	serine (or cysteine) peptidase inhibitor, clade I, member 1	Not regulated	Upregulated	
9	Mesangial cells	Tpm2	tropomyosin 2, beta	Not regulated	Upregulated	Striated Muscle Contraction
9	Mesangial cells	Wisp1	WNT1-inducible-signaling pathway protein 1	Not regulated	Down-regulated	
10	Collecting duct cells	Cadps2	Ca2+-dependent activator protein for secretion 2	Not regulated	Down-regulated	
10	Collecting duct cells	Col26a1	collagen, type XXVI, alpha 1	Not regulated	Upregulated	Collagen degradation
10	Collecting duct cells	Fxyd4	FXYD domain-containing ion transport regulator 4	Not regulated	Upregulated	Transport of small molecules
10	Collecting duct cells	Gm973	predicted gene 973	Not regulated	Upregulated	
10	Collecting duct cells	Kcne1	potassium voltage-gated channel, Isk-related subfamily, member 1	Not regulated	Down-regulated	Muscle contraction
10	Collecting duct cells	Me2	malic enzyme 2, NAD(+)-dependent, mitochondrial	Not regulated	Down-regulated	The citric acid (TCA) cycle and respiratory electron transport
10	Collecting duct cells	Mt2	metallothionein 2	Down-regulated	Down-regulated	
10	Collecting duct cells	Ptges	prostaglandin E synthase	Down-regulated	Down-regulated	Metabolism
10	Collecting duct cells	Rassf5	Ras association (RalGDS/AF-6) domain family member 5	Not regulated	Down-regulated	
10	Collecting duct cells	Slc9a4	solute carrier family 9 (sodium/hydrogen exchanger), member 4	Not regulated	Down-regulated	Transport of small molecules
10	Collecting duct cells	Spock1	sparc/osteonectin, cwcv and kazal-like domains proteoglycan 1	Not regulated	Down-regulated	

10	Collecting duct cells	Stc1	stanniocalcin 1	Not regulated	Upregulated	
10	Collecting duct cells	Sulf2	sulfatase 2	Not regulated	Down-regulated	
10	Collecting duct cells	Tsc22d1	TSC22 domain family, member 1	Down-regulated	Not regulated	
11	Thin ascending limb of of loop of Henle	Chaf1a	chromatin assembly factor 1, subunit A (p150)	Not regulated	Down-regulated	
11	Thin ascending limb of of loop of Henle	Galnt9	polypeptide N-acetylgalactosaminyltransferase 9	Not regulated	Upregulated	Metabolism of proteins
11	Thin ascending limb of of loop of Henle	Nefl	neurofilament, light polypeptide	Not regulated	Down-regulated	Neurotransmitter receptors and postsynaptic signal transmission
11	Thin ascending limb of of loop of Henle	Nsg2	neuron specific gene family member 2	Not regulated	Upregulated	
11	Thin ascending limb of of loop of Henle	Pappa2	pappalysin 2	Not regulated	Upregulated	Regulation of Insulin-like Growth Factor (IGF) transport and uptake by Insulin-like Growth Factor Binding Proteins (IGFBPs)
12	Non specific	Arhgdig	Rho GDP dissociation inhibitor (GDI) gamma	Not regulated	Down-regulated	Signal Transduction
12	Non specific	Dtx3	deltex 3, E3 ubiquitin ligase	Down-regulated	Not regulated	
12	Non specific	Fgf9	fibroblast growth factor 9	Upregulated	Upregulated	PI3K Cascade
12	Non specific	Gls	glutaminase	Not regulated	Down-regulated	Neurotransmitter release cycle
12	Non specific	Lrp6	low density lipoprotein receptor-related protein 6	Upregulated	Not regulated	Signal Transduction
12	Non specific	Sesn3	sestrin 3	Not regulated	Down-regulated	Generic Transcription Pathway
12	Non specific	Ung	uracil DNA glycosylase	Down-regulated	Not regulated	Cleavage of the damaged pyrimidine |Displacement of DNA glycosylase by APEX1

Supplementary Table S1
